# Study of the Preparation and Properties of Chrysin Binary Functional Monomer Molecularly Imprinted Polymers

**DOI:** 10.3390/polym14142771

**Published:** 2022-07-06

**Authors:** Long Li, Lanfu Li, Gege Cheng, Sentao Wei, Yaohui Wang, Qin Huang, Wei Wu, Xiuyu Liu, Guoning Chen

**Affiliations:** 1 School of Chemistry and Chemical Engineering, Guangxi Minzu University, Nanning 530006, China; lilong19980227@163.com (L.L.); a17876072393@163.com (L.L.); ggcheng2022@163.com (G.C.); a1184306866@163.com (S.W.); wwangyh970218@163.com (Y.W.); huangqin@gxun.edu.cn (Q.H.); 2 Key Laboratory of Chemistry and Engineering of Forest Products, State Ethnic Affairs Commission, Nanning 530006, China; 3 Guangxi Key Laboratory of Chemistry and Engineering of Forest Products, Nanning 530006, China; 4 Guangxi Collaborative Innovation Center for Chemistry and Engineering of Forest Products, Guangxi Minzu University, Nanning 530006, China; 5 Jihua Laboratory, 13 Nanpingxi Road, Foshan 528200, China; scutw.wei@gmail.com; 6 Guangxi Bossco Environmental Protection Technology Co., Ltd., Nanning 530007, China

**Keywords:** chrysin, molecular imprinting, adsorption performance, binary functional monomers

## Abstract

Chrysin is a natural bioactive molecule with various groups, and it has been a challenge to separate and enrich chrysin from natural products. Molecularly imprinted polymers have been widely used in the extraction of natural products, but the number and type of functional monomers limits the separation effect. The synergistic action of multiple functional monomers can improve the separation effect. In this paper, molecularly imprinted polymers (Bi-MIPs) were prepared using methacrylic acid and acrylamide as binary functional monomers for the separation and enrichment of chrysin. The Bi-MIPs were characterized using thermogravimetric analyzer (TGA), Fourier transform infrared spectroscopy (FT-IR) and scanning electron microscope (SEM). The performances of Bi-MIPs were assessed, which included adsorption isotherms, selective recognition and adsorption kinetics. The experimental results show that Bi-MIPs are shaped as a uniform sphere with an abundant pocket structure on its surface. The adsorption of chrysin on the Bi-MIPs followed a pseudo-second-order and adapted Langmuir–Freundlich isotherm models. The adsorption performance of the Bi-MIPs was determined at different temperatures, and the Bi-MIPs showed excellent adsorption performance at 30 °C. The initial decomposition temperature of the Bi-MIPs was 220 °C. After five times of adsorption and desorption, the adsorption performance of the Bi-MIPs decreased by only 7%. In contrast with single functional monomer molecularly imprinted polymers (Si-MIPs), the Bi-MIPs showed excellent specificity, with an imprinting factor of 1.54. The Bi-MIPs are promising materials in the separation and enrichment of chrysin for their high adsorption capacity, low cost and being environmentally friendly.

## 1. Introduction

Natural products have gained enormous popularity over the years as they are used in clinical settings [1]. They have made excellent contributions historically to drug development, and many of them have had profound effects on our lives [2]. Chrysin, chemically known as 5,7-dihydroxy flavone [3], is a kind of flavonoid compound with extensive pharmacological activity isolated from active ingredients of Chinese traditional medicine [4,5] and has great antioxidative [6] and anti-inflammatory effects [7]. Research has shown that chrysin inhibits tumor cell proliferation [8], induces tumor cell apoptosis [9], suppresses tumor angiogenesis [10] and circumvents drug resistance [11]. Therefore, studies on the extraction and identification of chrysin are of great value.

According to literature reviews, there are many methods for the separation of chrysin, including HPLC [12], column chromatography [13], solid-phase extraction (SPE) [14], gas chromatography–mass spectrometry(GC-MC) [15], macroporous resin [16] and other traditional methods. However, due to the problems of long extraction time and a large amount of solvent and waste, the instrumental methods are not suitable for industrial mass production applications. Although the traditional separation methods, such as extraction and precipitation, can be applied at a low cost, these approaches are restricted because of low recoveries and purities. At present, there is still a lack of efficient extraction methods of chrysin.

The molecularly imprinted polymer has been widely used for the separation and enrichment of active components from natural products [17,18,19,20,21]. Molecular imprinting is a technique for preparing polymers of desired and predetermined selectivity [22]. This polymer is a material with complementary spatial structure and functional group interaction with template molecule. The molecularly imprinted polymer has a strong affinity and recognition ability for the template molecule [23,24,25,26,27,28]. The method has excellent prospects for application in the field or market for easy and fast molecular identification. Due to their unique properties, molecularly imprinted polymers have been widely used in various applications, such as drug delivery [29], detection of viruses [30], chemical sensor [31,32], specific recognition of protein [33], chromatography [34], solid-phase extraction [35] and bioanalysis [36].

Generally speaking, most natural products have multiple active groups. Single functional monomers and template molecules are easily destroyed during hydrogen bond formation, which reduces adsorption capacity and separation factor and, thus, cannot be well separated and purified from traditional Chinese medicine. The synergistic effect of the two functional monomers is beneficial to the separation and purification of natural products. For example, Wan [37] selectively extracted myricetin from traditional Chinese medicine with an adsorption capacity of 10.58 mg/g using glycidyl methacrylate (GMA) and 4-vinylpyridine as bifocal monomers. Huan [38] used acrylamide and 2-vinylpyridine as bifocal monomers to prepare a solid-phase extraction column for the separation of rutin extract from traditional Chinese medicine, and the recovery rate was 85.93%, which was better than the traditional separation column. The calculation and practical experiments show that MIP synthesized from an acrylamide (AM) monomer has a higher specific factor and adsorption capacity [39]. Chrysin has a rigid benzene ring structure and contains both a hydroxyl group and an aldehyde group, belonging to polar flavonoids. The functional monomer methacrylic acid (MAA) was acidic and acrylamide (AM) neutral. Under the synergistic action of AM, the force of MAA on the hydrogen bonds, electrostatic and π–π stacking of chrysin increased, which is expected to improve the adsorption selectivity of molecularly imprinted polymers. Therefore, the strategy of multifunctional monomer is a valid synthetic option to synthesize imprinted materials for the template molecules of flavonoids with polar functional groups, such as chrysin.

In this paper, Bi-MIPs are used for the separation and enrichment chrysin for the first time. The Bi-MIPs were prepared by precipitation polymerization using methacrylic acid and acrylamide as functional monomers and ethylene glycol dimethacrylate as a crosslinking agent. The adsorption selectivity of the Bi-MIPs was evaluated by preparing Si-MIPs with methacrylic acid as a functional monomer as the control group. Furthermore, we also study the feasibility of the Bi-MIPs as effective sorptive materials for the dissociation and enrichment of chrysin. The equilibrium, kinetics and thermodynamics of the adsorption process were investigated to study the adsorption mechanism of chrysin on the Bi-MIPs. Thus, Bi-MIPs are presented for their promising output application toward the extraction of chrysin.

## 2. Materials and Methods

### 2.1. Materials

Chrysin, 2,2′-azobisisobutyronitrile (AIBN) and acrylamide (AM) were purchased from Shanghai Aladdin Biochemical Technology Co., Ltd. (Shanghai, China). Methyl alcohol and acetic acid were purchased from Chengdu Cologne Chemicals Co., Ltd. (Chengdu, China). Methacrylic acid (MAA) was purchased from Sinopharm Chemical Reagent Co., Ltd. (Shanghai, China). Ethylene glycol dimethacrylate (EGDMA) was purchased from Alfa Aesar (Qingdao, China). Chloramphenicol and oxytetracycline were purchased from Shanghai Maclin Biochemical Technology Co., Ltd. (Chengdu, China).

### 2.2. Preparation of Bi-MIPs, Bi-NIPs, Si-MIPs and Si-NIPs

**Figure 1:**
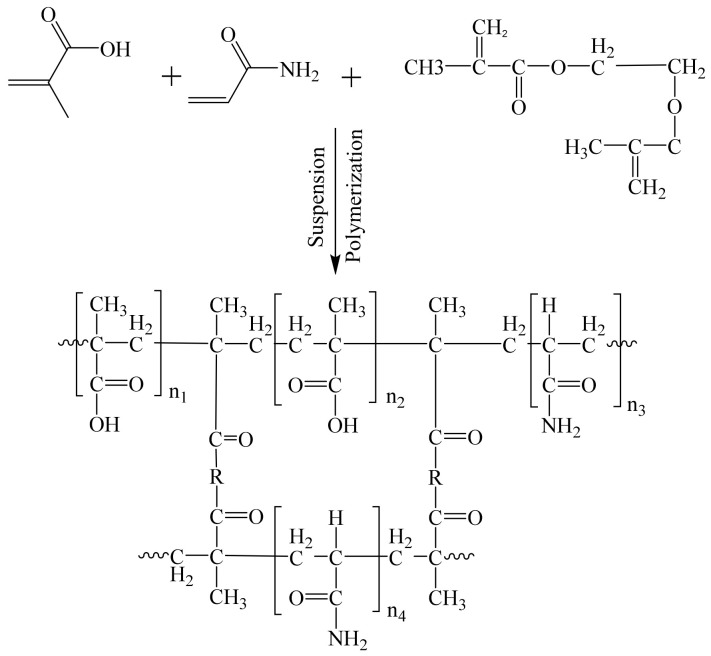
The scheme for preparing the Bi-MIPs (R is -O-CH_2_-CH_2_-O-).

The Bi-MIPs were synthesized by the precipitation polymerization method, using chrysin as template molecule, MAA and AM as function monomers, EGDMA as cross-linker and AIBN as initiator. Chrysin (0.3 mmol), MAA (2.4 mmol) and AM (0.4 mmol) were dissolved in 50 mL of methanol in a 250 mL three-necked round-bottomed flask. The function monomers (MAA (2.4 mmol) and AM (0.4 mmol)) interacted with the template molecule (chrysin) through intermolecular force to form a stable “pre-polymerization” complex. Then, the 50 mL of mixture solution of EGDMA (9.6 mmol), AIBN (0.1043 g) and methanol was added and dispersed under ultrasound. The mixture was heated followed by mechanical agitation (50 rpm) and heat-polymerized at 65 °C for 12 h. The synthesis process is shown in Figure 1. The Bi-MIPs were collected through vacuum filtration, and the unreacted monomers were removed by methanol: acetic acid (9:1, *v*/*v*) extraction for 24 h and methanol extraction for 12 h. The Bi-MIPs were dried under a vacuum at 50 °C for 12 h. The binary functional monomers molecularly non-imprinted polymer (Bi-NIP) microspheres were synthesized using the same procedure as the Bi-MIPs, but without the chrysin (template molecules). The Si-MIPs were synthesized following the same procedure without adding the AM. The single functional monomer molecularly non-imprinted polymers (Si-NIPs) were synthesized following the same procedure without adding the AM and chrysin.

### 2.3. Characterization of Bi-MIPs, Bi-NIPs, Si-MIPs and Si-NIPs

The analysis of the size and the morphology of the Bi-MIPs, Bi-NIPs, Si-MIPs and Si-NIPs were performed using field-emission scanning electron microscopy (SEM; Supra 55 Sapphire, Carl Zeiss, Jena, Germany). Thermogravimetric analysis (TGA) and differential thermogravimetric analysis (DTG) (STA 449 F5, NETZSCH-Gerätebau GmbH, Selb, Germany) were conducted to evaluate the thermal stabilities of the Bi-MIPs, Bi-NIPs, Si-MIPs and Si-NIPs under an N_2_ atmosphere at a heating rate of 10 K/min. The functional groups of the Bi-MIPs, Bi-NIPs, Si-MIPs and Si-NIPs were recorded by Fourier transform infrared spectrometer (MagnA-IR55, Thermo Fisher Scientific, Massachusetts, USA) in the range of 4000–400 cm^−1^. The chrysin solution was determined by UV-visible spectra (UV-2600 Shimadzu, Tokyo, Japan). The Bi-MIPs, Bi-NIPs, Si-MIPs and Si-NIPs were weighed by analytical balance with an accuracy of 0.1 mg (Practum124-1cn, Sartorius AG, Göttingen, Germany).

#### 2.3.1. Scanning Electron Microscopy (SEM)

The surface morphology of the Bi-MIPs, Bi-NIPs, Si-MIPs and Si-NIPs was determined by SEM analysis (SEM, Supra 55 Sapphire, Carl Zeiss Germany, Jena, Germany). The samples were evenly coated on the conductive adhesive of the sample sheet and then sprayed with gold for 0.5 h. The surface morphology of the Bi-MIPs, Bi-NIPs, Si-MIPs and Si-NIPs after the samples were sprayed with gold was observed by SEM under low vacuum conditions.

#### 2.3.2. Diameters of the Bi-MIPs, Bi-NIPs, Si-MIPs and Si-NIPs

The diameters of the Bi-MIPs, Bi-NIPs, Si-MIPs and Si-NIPs were measured from their SEM images using image analysis software (Image J). The diameter of more than 60 samples in each sample were measured. AD represents mean diameter ± standard deviation, and N represents sample quantity.

#### 2.3.3. Thermogravimetric Analysis (TGA)

The thermal stability of the Bi-MIPs, Bi-NIPs, Si-MIPs and Si-NIPs was determined by thermogravimetric analysis (TGA) (STA449F5, NETZSCH-Gerätebau GmbH, Selb, Germany). The samples were heated from 30 to 800 °C for thermal degradation under nitrogen protection at a rate of 10 °C/min [40].

#### 2.3.4. Nitrogen Adsorption/Desorption Analysis (BET)

The specific surface area and pore size of the samples were measured by the specific surface area and pore size analyzer (ASAP2460, Micromeritics, Georgia, USA). Nitrogen adsorption–desorption was measured at 90 °C for 12h under nitrogen protection [41].

### 2.4. Static Adsorption

#### 2.4.1. Adsorption Isotherm of Chrysin on Bi-MIPs, Bi-NIPs, Si-MIPs and Si-NIPs

To estimate the adsorption properties of Bi-MIPs, the Bi-MIPs were weighed (0.02 ± 0.0002 g) with an analytical balance and placed into a round of 50 mL conical flasks, and added into 20 mL of a chrysin methanol solution with different concentrations (0.2 mg/mL–1.4 mg/mL). The adsorption process of the Bi-MIPs was carried out for 5 h in an 80 rpm constant temperature oscillator at 25 °C. After the sorption experiments, the Bi-MIPs were separated from the mixed solution by a centrifuge and the concentration in the supernatant was obtained for UV-vis absorbance. The chrysin adsorption amount of Bi-MIPs was determined based on the following Equation (1):

(1) $$Q_{e}=\frac{{(C}_{0}{-C}_{e})\;\times \;V}{W}$$

where C_0_ (mg/mL) represents the initial concentration of chrysin in the solution, C_e_ (mg/mL) represents the equilibrium concentration of chrysin after adsorption, V (mL) represents the volume of the adsorbed chrysin solution and W (g) represents the mass of polymers in the adsorbed chrysin solution.

At the same time, to evaluate the adsorption capacity of Bi-NIPs, Si-MIPs and Si-MIPs. A total of 20 mg of Bi-NIPs, Si-MIPs or Si-MIPs was placed into a round of 50 mL conical flasks and added into 20 mL of chrysin solution with different concentrations (0.2 mg/mL–1.4 mg/mL). Under the same operating conditions of Bi-MIPs, the concentration of chrysin in the supernatant was analyzed using UV-vis absorbance. The equilibrium adsorption capacity was calculated using Equation (1).

#### 2.4.2. Adsorption Kinetics

To estimate the adsorption kinetics of Bi-MIPs on chrysin, the influence of the adsorption properties over time was studied. The Bi-MIPs were weighed (0.02 ± 0.0002 g) with an analytical balance and placed into a round of 50 mL conical flasks. After adding 20 mL of chrysin methanol solution, the Bi-MIPs were used to adsorb chrysin (1 mg/mL) in an 80 rpm constant temperature oscillator at 25 °C. Samples of 0.05 mL volume were taken at 15, 30, 45, 60, 75, 90, 105,120, 150, 180, 210, 240 and 300 min, and the concentration in the supernatant was obtained for UV-vis absorbance. The chrysin equilibrium adsorption amount of the Bi-MIPs was obtained based on the following Equation (2):

(2) $$Q_{t}=\frac{{(C}_{0}{-C}_{t})}{W}\;\times \;V$$

where C_0_ (mg/mL) represents the initial concentration of chrysin in the solution, C_t_ (mg/mL) represents the concentration of chrysin solution at time t (min), V (mL) represents the volume of adsorbed chrysin solution and W (g) represents the mass of polymers in the adsorbed chrysin solution.

At the same time, to evaluate the adsorption capacity of Bi-NIPs, Si-MIPs and Si-MIPs. Under the same operating conditions of the Bi-MIPs, the concentration of chrysin in the supernatant was analyzed using UV-vis absorbance. The equilibrium adsorption capacity was calculated using Equation (2).

#### 2.4.3. Adsorption Thermodynamics

To determine the adsorption temperature of chrysin on the Bi-MIPs, the effect of five different temperatures (10, 20, 30, 40 and 50 °C) on the adsorption capacity of chrysin by Bi-MIPs was evaluated. The Bi-MIPs were weighed (0.02 ± 0.0002 g) with an analytical balance and placed into a round of 50 mL conical flasks. After adding 20 mL of chrysin methanol solution, the Bi-MIPs were used to adsorb chrysin (1 mg/mL) in an 80 rpm constant temperature oscillator. The concentration of chrysin in the supernatant was analyzed using UV-vis absorbance. The equilibrium adsorption capacity was calculated using Equation (1). At the same time, to evaluate the adsorption temperature of chrysin on the Bi-NIPs, Si-MIPs and Si-MIPs, under the same operating conditions of the Bi-MIPs, the concentration of chrysin in the supernatant was analyzed using UV-vis absorbance. The equilibrium adsorption capacity was calculated using Equation (1).

### 2.5. Selective Adsorption

To evaluate the specific recognition ability of Bi-MIPs for chrysin, selective experiments were conducted with chloramphenicol and oxytetracycline as structural analogues. The Bi-MIPs were weighed (0.02 ± 0.0002 g) with an analytical balance and placed into a round of 50 mL conical flasks. After adding 20 mL of a solution of chrysin (1 mg/mL), chloramphenicol (1 mg/mL) and terramycin (1 mg/mL), it was adsorbed in an 80 rpm constant temperature oscillator at 25 °C. After the adsorption experiments, the Bi-MIPs were separated from the mixed solution by a centrifuge. The concentration in the supernatant was obtained by UV-vis absorbance. The selective adsorption ability of Bi-MIPs was obtained using the selectivity factors (α), which is shown in Equation (3).

(3) $$\alpha =\frac{Q_{\text{Bi-MIPs}}}{Q_{\text{Bi-NIPs}}}$$

### 2.6. Adsorption Reusability

After 20 mg Bi-MIP was adsorbed and balanced in 20 mL chrysin solution, the saturated adsorption sample was obtained by centrifugation. The Bi-MIPs were cleaned with a methanol solution for several times, and then dried and weighed. The processed Bi-MIPs were subjected to the adsorption test again. Under the same conditions, the adsorption–desorption cycle was performed 5 times, and the adsorption amount determined the concentration after adsorption each time and calculated the adsorption amount according to Formula (1).

## 3. Results and Discussion

### 3.1. Characterization of Bi-MIPs, Bi-NIPs, Si-MIPs and Si-NIPs

#### 3.1.1. SEM

**Figure 2:**
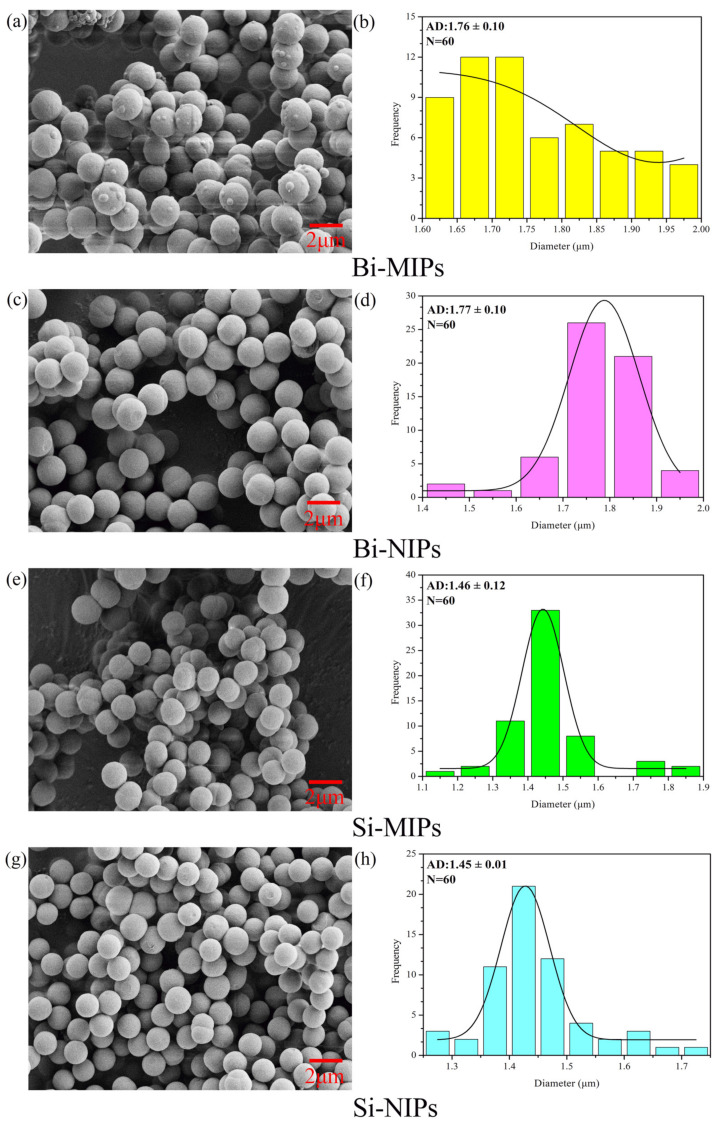
SEM, average particle size and particle size distribution images of Bi-MIPs (**a**,**b**), Bi-NIPs (**c**,**d**), Si-MIPs (**e**,**f**) and Si-NIPs (**g**,**h**).

The surfaces of Bi-MIPs, Bi-NIPs, Si-MIPs and Si-NIPs were analyzed using a scanning electron microscope. The most representative SEM images of all particles including Bi-MIPs, Bi-NIPs, Si-MIPs and Si-NIPs are shown in Figure 2. The SEM results show that were differences in the polymerization process in the presence of the template or without it. From Figure 2a–d, the as-synthesized polymers products present a regular and spherical structure with a diameter size of about 1–2 μm. The pore volume and surface area of the Bi-MIPs are 4.459 × 10^−3^ cm³/g and 2.8488 ± 0.1059 m^2^/g, respectively. The Bi-MIPs and Bi-NIPs particles are the same, but the Bi-MIPs have more surface folds, which can provide more adsorption sites [19,42]. The results show that the chrysin occupies a position on the polymer surface during the synthesis process. After elution with methanol and acetic acid, the surface of the Bi-MIPs becomes more wrinkled than that of Bi-NIPs. However, some clustered small particles remain on the surface of the Bi-MIPs, which shows that the template molecules had a remarkable effect on the shape and adsorption properties. This is due to the addition of template molecules in the synthesis of Bi-MIPs, which have a larger particle size and more surface adsorption sites due to the presence of template molecules that give the crosslinking agent and functional monomers a fuller skeleton. The Si-MIP particles are smaller than the Bi-MIP particles, which indicates that the number of functional monomers has a significant influence on the formation of particles and the final particle size. These results suggest that the Bi-MIPs are successfully synthesized.

#### 3.1.2. FT-IR

**Figure 3:**
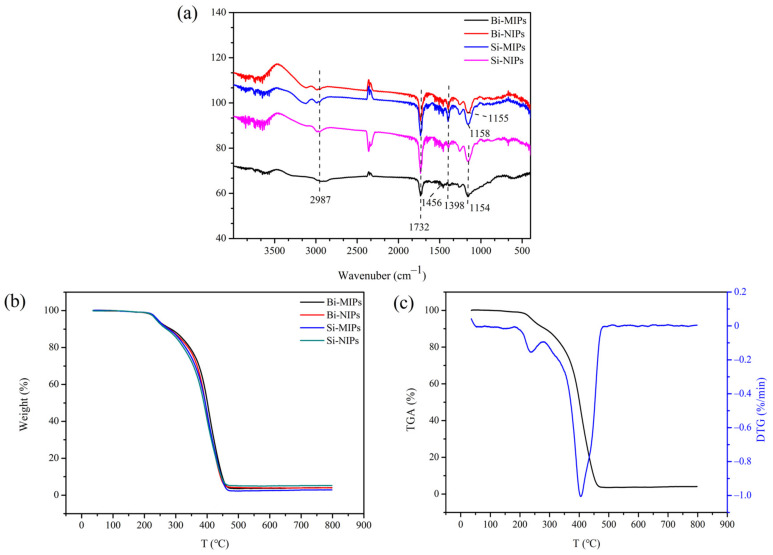
(**a**) FI-IR images of the Bi-MIPs, Bi-NIPs, Si-MIPs and Si-NIPs; (**b**) TGA images of the Bi-MIPs, Bi-NIPs, Si-MIPs and Si-NIPs; (**c**) TGA and DTG images of the Bi-MIPs.

The structures of the Bi-MIPs, Bi-NIPs, Si-MIPs and Si-NIPs were investigated by FT-IR spectroscopy, and the results are shown in Figure 3. As shown in Figure 3a, the Bi-MIPs, Bi-NIPs, Si-MIPs and Si-NIPs display a stretching vibration peak of -CH_2_- occurring at 2987 cm^−1^, indicating that MAA is contained in polymerization. The stretching vibration peak of the -NH- double bond appeared at 1456 cm^−1^, the single-bond vibration region of -CH appeared at 1398 cm^−1^, and the stretching vibration peak of -C=O occurred at 1732 cm^−1^ [42,43,44,45]. The carbonyl groups tend to have a higher vibration absorption, indicating the successful preparation of the Bi-MIPs, Bi-NIPs, Si-MIPs and Si-NIPs. The partial single-bond vibration absorption peak of -COC- was observed at 1154 cm^−1^ on the Bi-MIP curve, indicating the presence of EGDMA. At the same time, in the Bi-NIP, Si-MIP, and Si-NIP curves, partial single-bond vibration absorption peaks of -COC- were observed at about 1154 cm^−1^, indicating the presence of EGDMA, indicating that the polymer was successfully synthesized. The FT-IR of the Bi-MIPs and Bi-NIPs were almost the same. After removing the template molecule chrysin, the chemical structure and composition of the Bi-MIPs were the same as that of the Bi-NIPs. These results prove that the types of functional monomers have a certain influence on the synthesis of polymers, and the functional monomers play a role in the construction of adsorption pocket size.

#### 3.1.3. TGA

The thermal stabilities of the Bi-MIPs, Bi-NIPs, Si-MIPs and Si-NIPs were studied by TGA analysis, and the results are presented in Figure 3b,c. As shown in Figure 3b, the thermogravimetric analysis curves of the Bi-NIPs, Si-MIPs and Si-NIPs are the same as those of the Bi-MIPs without significant difference. The Bi-MIPs start to decompose at 220 °C, the temperature of the fastest decomposition rate occurs at 404 °C and the maximum decomposition temperature (Tmax) is 468 °C. The decomposition process is divided into two stages, including dehydration in the low-temperature zone (100–220 °C) and decomposition in the high-temperature zone (404–462 °C). The decomposition of the second stage is due to the decomposition of the crosslinker (EGDMA), indicating that the stability of the crosslinker is the main factor affecting the thermal stability of the polymers. The TGA results demonstrate that the Bi-MIPs have excellent thermal stability.

### 3.2. Static Adsorption Experiments

#### 3.2.1. Adsorption Isotherm

**Figure 4:**
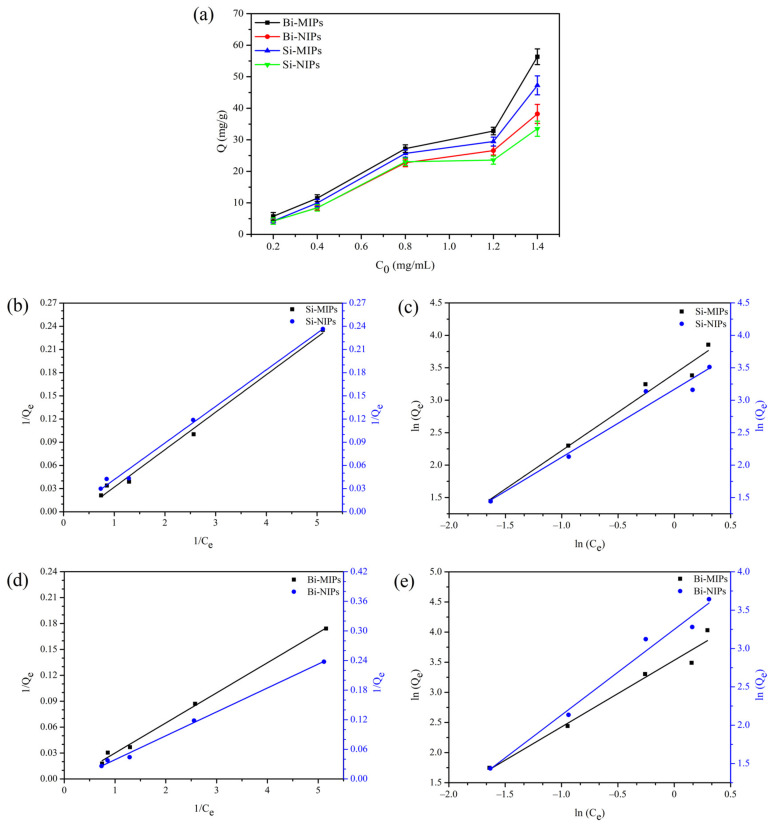
(**a**) Adsorption isotherm of the Bi-MIPs, Bi-NIPs, Si-MIPs and Si-NIPs; (**b**) Langmuir adsorption isotherm of the Si-MIPs and Si-NIPs; (**c**) Freundlich adsorption isotherm of the Si-MIPs and Si-NIPs; (**d**) Langmuir adsorption isotherm of the Bi-MIPs and Bi-NIPs; (**e**) Freundlich adsorption isotherm of the Bi-MIPs and Bi-NIPs.

The adsorption isotherms of chrysin on the Bi-MIPs, Bi-NIPs, Si-MIPs and Si-NIPs at (298 K) with chrysin concentrations of 0.2–1.4 mg/mL are presented in Figure 4. As shown in Figure 4a, the chrysin adsorption property for the Bi-MIPs, Bi-NIPs, Si-MIPs and Si-NIPs increased with increasing chrysin concentration. As the concentration increases, the adsorption difference increases. The Bi-MIPs have the highest adsorption capacity of chrysin, followed by Si-MIPs. These results indicate that the Bi-MIPs and Si-MIPs have specific cavities sizes and specific adsorption capacity for chrysin. By comparing Bi-MIPs, Bi-NIPs, Si-MIPs and Si-NIPs to the adsorption capacity of chrysin, the adsorption capacity of the binary functional monomers was better than that of the single functional monomer, which further indicates that the Bi-MIPs have an excellent application prospect.

To analyze the adsorption mechanism, Langmuir and Freundlich isotherm models were used to fit the experimental data when the adsorption process reached the adsorption equilibrium. The equations of these two models are as follows [46,47,48,49].

Langmuir isotherm equation:

(4) $$\frac{1}{Q_{e}}=\frac{1}{Q_{m}}+\frac{1}{K_{1}Q_{m}}\times \frac{1}{C_{e}}$$

Freundlich isotherm equation:

(5) $${\mathrm{lnQ}}_{e}{=\mathrm{lnk}}_{2}+\frac{1}{n}{\mathrm{lnC}}_{e}$$

where C_e_ represents the concentration of chrysin at the adsorption equilibrium (mg/mL); Q_e_ represents the chrysin adsorption amount for the Bi-MIPs, Bi-NIPs, Si-MIPs and Si-NIPs at equilibrium (mg/g); Q_m_ represents the maximum adsorption amount of monolayer coverage (mg/g); K_1_ represents the Langmuir constant (mL/mg); K_2_ represents the Freundlich constant; and 1/n represents the dimensionless Freundlich constant.

According to the Langmuir and Freundlich isotherm models, the experimental data were fitted and the parameters are shown in Table 1. The Bi-MIPs are illustrated by comparing the Langmuir and Freundlich equation correlation coefficients (the Langmuir and Freundlich correlation coefficients are 0.9953 and 0.9669, respectively). The isothermal adsorption curve of the Bi-MIPs is better represented by the Langmuir model. The Bi-NIPs, Si-MIPs and Si-NIPs were compared and analyzed by the Langmuir and Freundlich equation correlation coefficients (Langmuir and Freundlich correlation coefficients are 0.9946 and 0.9788, 0.9912 and 0.9736, 0.9905 and 0.9596, respectively), indicating that the Bi-NIPs, Si-MIPs and Si-NIPs with Bi-MIPs have the same degree of compatibility with Langmuir. The isothermal adsorption curves of the Bi-NIPs, Si-NIPs and Si-NIPs are better represented by the Langmuir model. These results indicate that the adsorption of chrysin on the Bi-MIPs occurs via monolayer adsorption, which shows that the Bi-MIPs can easily adsorb chrysin.

#### 3.2.2. Adsorption Kinetics

**Figure 5:**
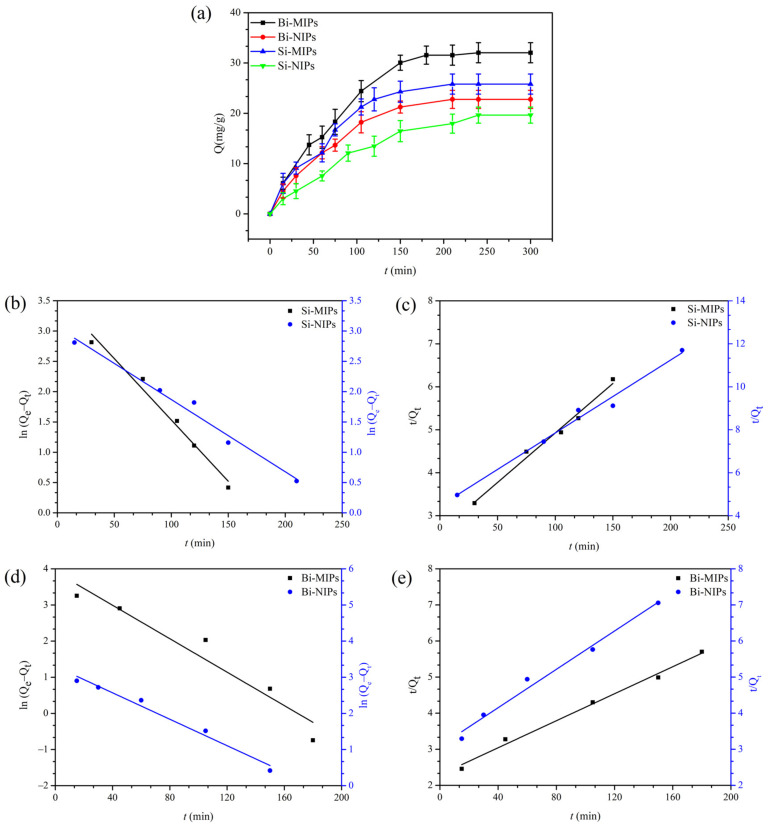
(**a**) Adsorption kinetics curves of the Bi-MIPs, Bi-NIPs, Si-MIPs and Si-NIPs; (**b**) PFO kinetic mode of the Si-MIPs and Si-NIPs; (**c**) PSO kinetic mode of the Si-MIPs and Si-NIPs; (**d**) PFO kinetic mode of the Bi-MIPs and Bi-NIPs; (**e**) PSO kinetic mode of the Bi-MIPs and Bi-NIPs.

Kinetic experiments were carried out on the Bi-MIPs, Bi-NIPs, Si-MIPs and Si-NIPs with a chrysin solution with an initial concentration of 1.0 mg/mL and the results are shown in Figure 5. The adsorption of chrysin on the Bi-MIPs showed excellent characteristics of the adsorption kinetics. The adsorption capability is increased with the increase in the adsorption time, and the adsorption rate decreased gradually with increasing adsorption time. At any time, the Bi-MIPs have the highest adsorption capacity of chrysin compared with the Bi-NIPs. As for the Bi-MIPs, the adsorption process can be divided into the fast adsorption stage (0–105 min) and the slow adsorption stage (105–240 min). The adsorption capacity in the fast adsorption stage accounted for 76% of the equilibrium adsorption capacity. As for the Si-MIPs, the adsorption capacity in the fast adsorption stage (0–75 min) accounted for 64.7% of the equilibrium adsorption capacity. The chrysin absorption capacity of Bi-MIPs reached equilibrium after 240 min, which indicates that the specific cavities of the adsorbent formed by binary functional monomers promote the adsorption effect. At the same time, the chrysin absorption capacity of the Bi-MIPs, Bi-NIPs, Si-MIPs, Si-NIPs and MIPs exceeds the NIPs, which further indicates that Si-MIPs and Bi-MIPs have successfully synthesized imprinted pores.

The pseudo-first-order (PFO) and pseudo-second-order (PSO) kinetic models were used to investigate the kinetic behaviors of the Bi-MIPs, Bi-NIPs, Si-MIPs and Si-NIPs during chrysin adsorption. The kinetic data of different initial melanoidin concentrations were fitted using the following models [50,51,52,53,54].

PFO kinetic equation:

(6) $$\ln\left(Q_{e}-Q_{t}\right){=\mathrm{lnQ}}_{e}-\frac{k_{3}}{2.303}$$

PSO kinetic equation:

(7) $$\frac{t}{Q_{t}}=\frac{1}{k_{4}Q_{e}^{2}}+\frac{1}{Q_{e}}$$

where k_3_ (1/min) represents the rate constant of PFO kinetic adsorption, k_4_ (mg/(g·min)) represents the rate constant of PSO kinetic adsorption, Q_t_ represents the amounts of chrysin adsorbed (mg/g) at time t, and Q_e_ represents the amounts’ equilibrium time.

The experimental data were fitted to the PFO kinetic and PSO kinetic to obtain the corresponding fitting curves (Figure 5) and kinetic parameters (Table 2). It is known from Table 2 that the correlation coefficient (R^2^) values of the PSO and PFO kinetic models of the Bi-MIPs are R^2^ = 0.9903 and R^2^ = 0.9153, respectively. The PSO kinetic model creates better experiments with the adsorption behavior of chrysin onto the Bi-MIPs than the PFO kinetic model; this phenomenon also indicates that chemisorption is the principal mechanism involved in the sorption process. The results indicate that the Bi-MIPs are beneficial to the adsorption of chrysin, and these results further prove the potential applications in the separation of chrysin by the Bi-MIPs.

#### 3.2.3. Adsorption Thermodynamics

**Figure 6:**
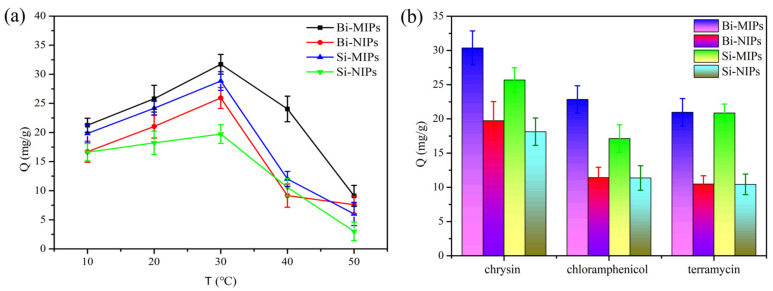
(**a**) Thermodynamic curves of the Bi-MIPs, Bi-NIPs, Si-MIPs and Si-NIPs; (**b**) Selective adsorption of the Bi-MIPs, Bi-NIPs, Si-MIPs and Si-NIPs.

Adsorption isotherm experiments were performed at the same initial chrysin solution and at different temperatures of 10, 20, 30, 40 and 50 °C. The fitting results of the adsorption isotherms are presented in Figure 6. As shown in Figure 6a, the adsorption ability of chrysin by the Bi-MIPs is the highest, followed by the Si-MIPs. This is due to the specific cavities of the Bi-MIPs and Si-MIPs, which have specific adsorption on chrysin.

With the temperature increase from 10 to 30 °C, the absorption capacity of the adsorbents increased, which due to increases in temperature accelerate the movement of the molecules in a methanol solution. Therefore, the probability of albumin binding to the adsorbent adsorption site is enhanced. As the temperature increased from 30 to 50 °C, the Q_e_ of chrysin on the adsorbents decreased. Due to the hydrogen bonds of the adsorbents being broken with the increase in temperature, the adsorption capacity of chrysin by the adsorbents is weakened. From the point of view of economic and industrial applications, 30 °C is chosen as the optimum temperature. The energy consumption of the adsorption process is reduced, which builds the foundation for the large-scale extraction and separation of the binary functional monomer polymers in natural products.

### 3.3. Adsorption Selectivity

To further investigate the selectivity of the Bi-MIPs for chrysin, chloramphenicol and oxytetracycline were chosen as comparative substrates in the selective adsorption test. The results are shown in Figure 6b; the Bi-MIPs have the highest adsorption capacity for chrysin, followed by the Si-MIPs. This is due to the specific pockets of Bi-MIPs and Si-MIPs, which have specific adsorption on chrysin. The Bi-MIPs and Si-MIPs have electrostatic adsorption on chloramphenicol and oxytetracycline. The imprinting factor (Q_Bi-MIPs_/Q_Bi-NIPs_) of the Bi-MIPs is 1.54, and the imprinting factor (Q_Si-MIPs_/Q_Si-NIPs_) of the Si-MIPs is 1.42. These results indicate that, under the synergistic action of AM, the force of MAA on the hydrogen bonds, electrostatic and π-π stacking of chrysin increased [37,38,39], and the adsorption selectivity of molecularly imprinted polymers is improved. The selectivity of binary functional monomers in the separation and extraction of chrysin further proves that the Bi-MIPs have broad application prospects in the extraction and separation of natural products.

### 3.4. Adsorption Reusability

**Figure 7:**
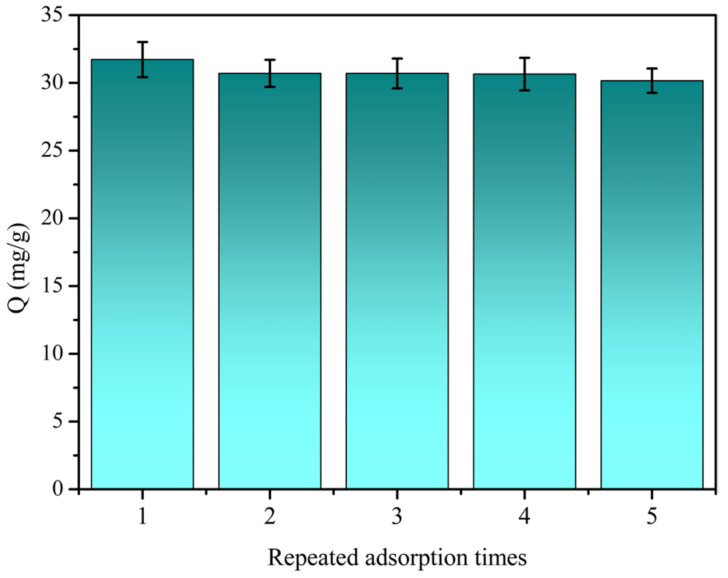
Regeneration rebinding performance of the Bi-MIPs.

To evaluate the capacity of the Bi-MIPs to be regenerated and reused, the adsorption performance after repeated cycles was investigated. Multiple adsorptions and desorption experiments were carried out using a polymer chrysin solution, and the results are shown in Figure 7. After five times of adsorption and desorption, the remaining solid mass of the sample is 0.0402, 0.0356, 0.0356, 0.0317, and 0.0302. The solid loss rate is 24.88%. After being recycled and reused, the Bi-MIPs only lost 4.92% of adsorption capacity. This decrease may be attributed to the reduction in active binding loci following the regeneration and inadequate desorption of the adsorbed chrysin molecules. The results suggest that the Bi-MIPs exhibited excellent adsorption capability in all five cycles. At the same time, we should further consider how to improve the recovery efficiency of the Bi-MIPs.

## 4. Conclusions

The Bi-MIPs were prepared by precipitation polymerization using methacrylic acid and acrylamide as functional monomers and ethylene glycol dimethacrylate as a crosslinking agent. Their physicochemical properties and chemical structures were analyzed and characterized. The Bi-MIP has excellent adsorption performance for chrysin in methanol solution and has a larger specific surface area and thermodynamic properties. The adsorption of chrysin on the Bi-MIPs followed the PSO kinetic model and Langmuir isothermal model, and the adsorption process was dominated by homogeneous monolayer adsorption. Compared with the Si-MIPs, the Bi-MIPs showed excellent adsorption performance and specificity in the adsorption process of chrysin and its analogs. At the same time, the Bi-MIPs showed excellent adsorption performance in multiple cycles and were conducive to the extraction and purification of an organic solvent-soluble bioactive component from natural products. Under the synergistic action of AM, the force of MAA on the hydrogen bonds, electrostatic and π-π stacking of chrysin increased. This synthetic method is green, efficient, eco-friendly and cost-effective. Thus, this paper provides new ideas and methods for the synthesis of high adsorption performance, green and safe molecular-imprinted polymers and build the foundation for the large-scale extraction and separation of the Bi-MIPs in chrysin.

## Figures and Tables

**Table 1:** Parameters of the Langmuir and Freundlich adsorption models.

Samples	Langmuir Isotherm			Freundlich Isotherm		
	K_1_ (mL mg^−1^)	R^2^	Q_m_ (mg g^−1^)	K_2_ (mL mg^−1^)	R^2^	1/n
Bi-MIPs	0.1370	0.9953	209.64	34.20	0.9669	1.106
Bi-NIPs	0.1935	0.9946	106.72	25.77	0.9788	1.116
Si-MIPs	0.1193	0.9912	60.350	30.20	0.9736	1.183
Si-NIPs	0.3417	0.9905	176.99	23.75	0.9596	1.046

**Table 2:** Kinetic data of the PFO and PSO kinetic models.

Samples	PFO Kinetic		PSO Kinetic	
	K_3_ (min^−1^)	R^2^	K_4_ (g mg^−1^ min^−1^)	R^2^
Bi-MIPs	0.0533	0.9153	0.00015	0.9903
Bi-NIPs	0.0421	0.9746	0.00023	0.9816
Si-MIPs	0.0466	0.9751	0.00020	0.9841
Si-NIPs	0.0275	0.9750	0.00026	0.9808

## References

[1] Ong S., Shanmugam M., Fan L., Fraser S., Arfuso F., Ahn K., Sethi G., Bishayee A. (2019). Focus on Formononetin: Anticancer Potential and Molecular Targets. Cancers.

[2] Zheng X., Wu F., Lin X., Shen L., Feng Y. (2018). Developments in drug delivery of bioactive alkaloids derived from traditional Chinese medicine. Drug Deliv..

[3] Subramanya S.B., Venkataraman B., Meeran M.F.N., Goyal S.N., Patil C.R., Ojha S. (2018). Therapeutic Potential of Plants and Plant Derived Phytochemicals against Acetaminophen-Induced Liver Injury. Int. J. Mol. Sci..

[4] Ramesh P., Rao V.S., Hong Y.-A., Reddy P.M. (2019). Molecular Design, Synthesis, and Biological Evaluation of 2-Hydroxy-3-Chrysino Dithiocarbamate Derivatives. Molecules.

[5] Al-Oudat B., Ramapuram H., Malla S., Audat S., Hussein N., Len J., Kumari S., Bedi M., Ashby C., Tiwari A. (2020). Novel Chrysin-De-Allyl PAC-1 Hybrid Analogues as Anticancer Compounds: Design, Synthesis, and Biological Evaluation. Molecules.

[6] Liu C., Kou X., Wang X. (2021). Novel chrysin derivatives as hidden multifunctional agents for anti-Alzheimer’s disease: Design, synthesis and in vitro evaluation. Eur. J. Pharm. Sci..

[7] Yu C.-H., Suh B., Shin I., Kim E.-H., Kim D., Shin Y.-J., Chang S.-Y., Baek S.-H., Kim H., Bae O.-N. (2019). Inhibitory Effects of a Novel Chrysin-Derivative, CPD 6, on Acute and Chronic Skin Inflammation. Int. J. Mol. Sci..

[8] Maruhashi R., Eguchi H., Akizuki R., Hamada S., Furuta T., Matsunaga T., Endo S., Ichihara K., Ikari A. (2019). Chrysin enhances anticancer drug-induced toxicity mediated by the reduction of claudin-1 and 11 expression in a spheroid culture model of lung squamous cell carcinoma cells. Sci. Rep..

[9] Xu M., Shi H., Liu D. (2019). Chrysin protects against renal ischemia reperfusion induced tubular cell apoptosis and inflammation in mice. Exp. Ther. Med..

[10] Schindler R.M.R. (2006). Flavonoids and vitamin E reduce the release of the angiogenic peptide vascular endothelial growth factor from human tumor cells. J. Nutr..

[11] Gilles Comte J.B.D., Bayet C. (2001). C-Isoprenylation of Flavonoids Enhances Binding Affinity toward P-Glycoprotein and Modulation of Cancer Cell Chemoresistance. J. Med. Chem..

[12] Gharari Z., Bagheri K., Danafar H., Sharafi A. (2019). Simultaneous determination of baicalein, chrysin and wogonin in four Iranian Scutellaria species by high performance liquid chromatography. J. Appl. Res. Med. Aromat. Plants.

[13] Gu Y., Chen X., Wang R. (2019). Comparative two-dimensional HepG2 and L02/cell membrane chromatography/C18/time-of-flight mass spectrometry for screening selective anti-hepatoma components from Scutellariae Radix. J. Pharm. Biomed. Anal..

[14] Mohammad Reza H., Saman N., Kamyar K. (2009). Determination of Flavonoid Markers in Honey with SPE and LC using Experimental Design. Chromatographia.

[15] Maciejewicz W., Daniewski M., Bal K., Markowski W. (2001). GC-MS identification of the flavonoid aglycones isolated from propolis. Chromatographia.

[16] Zhang Y., Wang B., Jia Z., Scarlett C.J., Sheng Z. (2018). Adsorption/desorption characteristics and enrichment of quercetin, luteolin and apigenin from Flos populi using macroporous resin. Rev. Bras. Farm..

[17] Ma Y., Wang H., Guo M. (2019). Stainless Steel Wire Mesh Supported Molecularly Imprinted Composite Membranes for Selective Separation of Ebracteolata Compound B from Euphorbia fischeriana. Molecules.

[18] Kamaruzaman S., Nasir N.M., Faudzi S.M.M., Yahaya N., Hanapi N.S.M., Ibrahim W.N.W. (2021). Solid-Phase Extraction of Active Compounds from Natural Products by Molecularly Imprinted Polymers: Synthesis and Extraction Parameters. Polymers.

[19] Ariani M.D., Zuhrotun A., Manesiotis P., Hasanah A.N. (2022). Magnetic Molecularly Imprinted Polymers: An Update on Their Use in the Separation of Active Compounds from Natural Products. Polymers.

[20] Sun X., Zhang Y., Zhou Y., Lian X., Yan L., Pan T., Jin T., Xie H., Liang Z., Qiu W. (2021). NPCDR: Natural product-based drug combination and its disease-specific molecular regulation. Nucleic Acids Res..

[21] Wang T., Wang Q., Guo Q., Li P., Yang H. (2021). A hydrophobic deep eutectic solvents-based integrated method for efficient and green extraction and recovery of natural products from Rosmarinus officinalis leaves, Ginkgo biloba leaves and Salvia miltiorrhiza roots. Food Chem..

[22] Luliński P., Maciejewska D. (2009). Examination of Imprinting Process with Molsidomine as a Template. Molecules.

[23] Peeters M.M., Van Grinsven B., Foster C.W., Cleij T.J., Banks C.E. (2016). Introducing Thermal Wave Transport Analysis (TWTA): A Thermal Technique for Dopamine Detection by Screen-Printed Electrodes Functionalized with Molecularly Imprinted Polymer (MIP) Particles. Molecules.

[24] Zhang W., Li Q., Cong J., Wei B., Wang S. (2018). Mechanism Analysis of Selective Adsorption and Specific Recognition by Molecularly Imprinted Polymers of Ginsenoside Re. Polymers.

[25] Chen J., Bai L.-Y., Liu K.-F., Liu R.-Q., Zhang Y.-P. (2014). Atrazine Molecular Imprinted Polymers: Comparative Analysis by Far-Infrared and Ultraviolet Induced Polymerization. Int. J. Mol. Sci..

[26] Si Z., Yu P., Dong Y., Lu Y., Tan Z., Yu X., Zhao R., Yan Y. (2019). Thermo-Responsive Molecularly Imprinted Hydrogels for Selective Adsorption and Controlled Release of Phenol From Aqueous Solution. Front. Chem..

[27] Xing R., Ma Y., Wang Y., Wen Y., Liu Z. (2018). Specific recognition of proteins and peptides *via* controllable oriented surface imprinting of boronate affinity-anchored epitopes. Chem. Sci..

[28] Piletska E., Yawer H., Canfarotta F., Moczko E., Smolinska-Kempisty K., Piletsky S.S., Guerreiro A., Whitcombe M.J., Piletsky S.A. (2017). Biomimetic Silica Nanoparticles Prepared by a Combination of Solid-Phase Imprinting and Ostwald Ripening. Sci. Rep..

[29] Liu R., Poma A. (2021). Advances in Molecularly Imprinted Polymers as Drug Delivery Systems. Molecules.

[30] Soufi G.J., Iravani S., Varma R.S. (2021). Molecularly imprinted polymers for the detection of viruses: Challenges and opportunities. Analyst.

[31] Ramanavicius S., Jagminas A., Ramanavicius A. (2021). Advances in Molecularly Imprinted Polymers Based Affinity Sensors (Review). Polymers.

[32] Zhao H., Gao W.-C., Li Q., Khan M.R., Hu G.-H., Liu Y., Wu W., Huang C.-X., Li R.K. (2022). Recent advances in superhydrophobic polyurethane: Preparations and applications. Adv. Colloid Interface Sci..

[33] He X., Wang Y., Li H. (2021). Specific recognition of protein by deep eutectic solvent-based magnetic beta-cyclodextrin molecularly imprinted polymer. Mikrochim. Acta.

[34] Xie L., Xiao N., Li L., Xie X., Li Y. (2020). Theoretical Insight into the Interaction between Chloramphenicol and Functional Monomer (Methacrylic Acid) in Molecularly Imprinted Polymers. Int. J. Mol. Sci..

[35] Song X., Li J., Wang J., Chen L. (2009). Quercetin molecularly imprinted polymers: Preparation, recognition characteristics and properties as sorbent for solid-phase extraction. Talanta.

[36] Sobiech M., Luliński P., Wieczorek P.P. (2021). Quantum and carbon dots conjugated molecularly imprinted polymers as advanced nanomaterials for selective recognition of analytes in environmental, food and biomedical applications. TrAC Trends Anal. Chem..

[37] Wan Y., Wang M., Fu Q., Wang L., Wang D., Zhang K., Xia Z., Gao D. (2018). Novel dual functional monomers based molecularly imprinted polymers for selective extraction of myricetin from herbal medicines. J. Chromatogr. B.

[38] Zeng H., Wang Y., Liu X., Kong J., Nie C. (2012). Preparation of molecular imprinted polymers using bi-functional monomer and bi-crosslinker for solid-phase extraction of rutin. Talanta.

[39] Thach U.D., Thi H.H.N., Pham T.D., Mai H.D., Nhu-Trang T.-T. (2021). Synergetic Effect of Dual Functional Monomers in Molecularly Imprinted Polymer Preparation for Selective Solid Phase Extraction of Ciprofloxacin. Polymers.

[40] Gao W.-C., Wu W., Chen C.-Z., Zhao H., Liu Y., Li Q., Huang C.-X., Hu G.-H., Wang S.-F., Shi D. (2021). Design of a Superhydrophobic Strain Sensor with a Multilayer Structure for Human Motion Monitoring. ACS Appl. Mater. Interfaces.

[41] Jiang Y., Wang Z., Zhou L., Jiang S., Liu X., Zhao H., Huang Q., Wang L., Chen G., Wang S. (2022). Highly efficient and selective modification of lignin towards optically designable and multifunctional lignocellulose nanopaper for green light-management applications. Int. J. Biol. Macromol..

[42] Li L., Liu X., Li L. (2022). Preparation of Rosin-Based Composite Membranes and Study of Their Dencichine Adsorption Properties. Polymers.

[43] Zahara S., Minhas M.A., Shaikh H., Ali M.S., Bhanger M.I., Malik M.I. (2021). Molecular imprinting-based extraction of rosmarinic acid from Salvia hypoleuca extract. React. Funct. Polym..

[44] Jiang Y., Wang Z., Liu X., Yang Q., Huang Q., Wang L., Dai Y., Qin C., Wang S. (2020). Highly Transparent, UV-Shielding, and Water-Resistant Lignocellulose Nanopaper from Agro-Industrial Waste for Green Optoelectronics. ACS Sustain. Chem. Eng..

[45] Cheng B.-X., Gao W.-C., Ren X.-M., Ouyang X.-Y., Zhao Y., Zhao H., Wu W., Huang C.-X., Liu Y., Liu X.-Y. (2022). A review of microphase separation of polyurethane: Characterization and applications. Polym. Test..

[46] Azizi S., Shahri M.M., Mohamad R. (2017). Green Synthesis of Zinc Oxide Nanoparticles for Enhanced Adsorption of Lead Ions from Aqueous Solutions: Equilibrium, Kinetic and Thermodynamic Studies. Molecules.

[47] Bagbi Y., Sarswat A., Mohan D., Pandey A., Solanki P.R. (2017). Lead and Chromium Adsorption from Water using L-Cysteine Functionalized Magnetite (Fe_3_O_4_) Nanoparticles. Sci. Rep..

[48] Moon G.H., Jung Y., Shin B. (2020). On-Chip Chemiresistive Sensor Array for On-Road NO x Monitoring with Quantification. Adv. Sci..

[49] Pham T., Bui T.T., Nguyen V.T., Van Bui T.K., Tran T.T., Phan Q.C., Hoang T.H. (2018). Adsorption of Polyelectrolyte onto Nanosilica Synthesized from Rice Husk: Characteristics, Mechanisms, and Application for Antibiotic Removal. Polymers.

[50] Liu H., Zhang F., Peng Z. (2019). Adsorption mechanism of Cr(VI) onto GO/PAMAMs composites. Sci. Rep..

[51] Duan C., Zhang Y., Li J., Kang L., Xie Y., Qiao W., Zhu C., Luo H. (2020). Rapid Room-Temperature Preparation of Hierarchically Porous Metal–Organic Frameworks for Efficient Uranium Removal from Aqueous Solutions. Nanomaterials.

[52] Ni X., Zhao Z., Li Z., Li Q. (2021). The adsorptive behaviour of kaolinite to sodium dodecyl benzene sulphonate and the structural variation of kaolinite. Sci. Rep..

[53] Dasgupta-Schubert N., Tiwari D.K., Cendejas L.M.V. (2016). Comment on ‘Carbon and fullerene nanomaterials in plant system’. J. Nanobiotechnol..

[54] Raibaut L., Cargoët M., Ollivier N., Chang Y.M., Drobecq H., Boll E., Desmet R., Monbaliu J.-C.M., Melnyk O. (2016). Accelerating chemoselective peptide bond formation using bis(2-selenylethyl)amido peptide selenoester surrogates. Chem. Sci..

